# HPV and Cervical Cancer—Biology, Prevention, and Treatment Updates

**DOI:** 10.3390/curroncol32030122

**Published:** 2025-02-22

**Authors:** Emilia Włoszek, Kamila Krupa, Eliza Skrok, Michał Piotr Budzik, Andrzej Deptała, Anna Badowska-Kozakiewicz

**Affiliations:** 1 Students’ Scientific Organization of Cancer Cell Biology, Department of Oncology Propaedeutics, Medical University of Warsaw, 02-091 Warsaw, Poland; kamila.krupa@student.wum.edu.pl (K.K.); s085306@student.wum.edu.pl (E.S.); 2 Department of Oncology Propaedeutics, Medical University of Warsaw, 02-091 Warsaw, Poland; michal.budzik@wum.edu.pl (M.P.B.); andrzej.deptala@wum.edu.pl (A.D.); abadowska@wum.edu.pl (A.B.-K.)

**Keywords:** human papillomavirus, HPV, cervical cancer, vaccination, screening, systemic therapy

## Abstract

One of the most significant breakthroughs in cancer research has been the identification of persistent infection with certain human papillomaviruses (HPV) genotypes as the cause of cervical cancer. Since then, a range of diagnostic and therapeutic methods has been developed based on this discovery. This article aims to describe the latest updates in the biology, prevention, and treatment of HPV-related cervical cancer. The current state of knowledge regarding vaccinations, diagnostic tests, and cervical cancer therapies is presented. The latest WHO guidelines on vaccinations are presented, as well as announcements of upcoming changes. The final part of the article summarizes promising new diagnostic and treatment methods, as well as perspectives and the latest research findings on self-administered diagnostic tests, the use of therapeutic vaccines, and circulating cell-free DNA in diagnosis. Despite the significant progress made in recent years, the strategy based on vaccination and testing remains the cornerstone in the fight against HPV-related cervical cancer.

## 1. Introduction

Papillomaviruses are small, double-stranded DNA viruses that specifically infect squamous epithelia or cells capable of squamous maturation. These viruses are strictly species-specific, meaning that human papillomaviruses (HPVs) exclusively infect humans. They exhibit remarkable tissue tropism, completing their infectious cycle only in fully differentiated squamous epithelium. Over the past 25 years, one of the most significant breakthroughs in cancer research has been the identification of persistent infection with certain HPV genotypes as the cause of cervical cancer. Comprehensive virological, molecular, clinical, and epidemiological studies have provided definitive evidence that cervical cancer results from prolonged, unresolved infection by specific HPV genotypes. Consequently, it is now understood that cervical cancer is the outcome of a viral infection. This recognition highlights vaccination as a vital strategy for the primary prevention of cancers and other diseases associated with HPV. Considering the findings of the research conducted in recent years, HPV infection may be a contributing factor in the development of many other types of cancer. The aim of this paper is to discuss the current state of knowledge about the HPV virus, present the latest discoveries in this field, and explore potential future possibilities related to diagnosis and treatment. PubMed and Google Scholar databases were used to collect data for this review using the following phrases: HPV epidemiology, HPV classification, HPV cycle, HPV pathogenesis, HPV screening, colposcopy, microbiota dysbiosis and HPV, HPV vaccine, HPV infection, HPV related cancers, cervical cancer screening, cervical cancer treatment, advanced cervical cancer, and HPV E6 E7.

## 2. Epidemiology

Chesson et al. estimated the lifetime probability of human papillomavirus (HPV) infection in the United States before it was introduced to HPV vaccination. The analysis revealed that over 80% of men and women would have HPV by age 45. Among individuals with at least one opposite-sex partner, 85% of women and 91% of men were projected to contract HPV during their lifetime [1]. The metanalysis from 2010, encompassing 194 studies, including about one million women tested for cervical HPV infection, showed that the most common types are HPV16, 18, 52, 31, 58, 39, 51, and 56, but the prevalence differs among the regions. Sub-Saharan Africa had the highest prevalence (24.0%), especially Eastern Africa (33.6%). The infection is more prevalent in poor nations than in industrialized ones, according to the geographic distribution. However, regardless of development level, West Asia (1.7%) exhibits the lowest prevalence, whereas Eastern Europe (21.4%) exhibits high prevalence [2]. North America has the lowest genotypic diversity, while Asia displays the highest [3]. Moreover, the latest meta-analysis showed that genital HPV infection prevalence is high in men over the age of 15. Globally, nearly one in three men are infected with HPV of any type, and approximately one in five men carry one or more high-risk (HR) HPV types [4].

## 3. Classification of HPV Viruses

The classification of the HPV viruses is based on differences in DNA sequence. There are five genera of HPV: alpha, beta, gamma, mu, and nu, which are divided into HR-HPV and low-risk HPV (LR-HPV) subtypes [5]. Currently, there are at least 448 types of HPV, 15 of which have been classified as carcinogenic: HPV16, 18, 31, 33, 35, 39, 45, 51, 52, 56, 58, 59, 68, 73, and 82 [6,7]. Types 26, 53, and 66 are considered potentially carcinogenic [7]. HPV16 and HPV18 exhibit the highest oncogenic potential; nevertheless, regional genetic diversity influences their pathogenicity [8]. Based on comparisons of the HPV16 long control region (LCR) sequences, we can distinguish five groups: European (E), Asian (As), Asian-American (AA), African 1 (Af-1), and African 2 (Af-2) [3].

## 4. Risk Factors

While barrier contraception methods, e.g. condoms, are successful in minimizing the transmission of numerous sexually transmitted infections, they are not entirely effective against HPV, as the virus can also be transferred via skin-to-skin contact or by contact between infected fingers and genitalia [9]. In addition, C. trachomatis may increase the risk of HPV infection and promote virus persistence [10,11]. Other risk factors include gender, young age, and the number of sexual partners [9,12]. Intercourse with a new sexual partner carries an increased risk of viral infection and, potentially, cancer development [13,14].

Women are more susceptible to infection with the HPV. The frequency of HPV detection occurs in two peaks. The most infected are girls in puberty and women under 20 years of age. Detection of HPV, especially LR-HPV, also concerns elderly women over 55 years of age, which may be related to the persistence or reactivation of a previously acquired infection, rather than new or recent infections [15,16]. An increasing number of full-term pregnancies is associated with a higher risk of invasive cervical carcinoma and chronic HPV infection. This association may be due to pregnancy-induced immunosuppression, which may favor the HPV infection or enhance its carcinogenic potential [17,18].

Children may become infected with HPV through contact between a parent’s wart, present on the hand, and the child’s skin while changing a diaper or cleaning the anogenital area [19,20]. There is also a risk of infection with the virus in the perinatal period, because the same type of HPV has been detected in both mothers and newborns [21]. If the mother has a genital HPV infection, the fetus can contract the virus through micro-tears in the fetal membranes or the placenta [19]. Since HPVs do not induce viraemia, it is unlikely that the virus is spread through breast milk [22].

New studies show that tobacco smoking promotes the progression of HR-HPV-induced lesions to precancerous and squamous cell carcinomas of the head and neck, cervix, anus, and penis [23,24,25]. Compounds present in cigarette smoke, of which at least 60 are oncogenic in humans, may contribute to increased expression of the virus in cells [26]. Whether oral hormonal contraception increases the progression of HPV infection remains controversial, and further analysis is therefore necessary [27]. The latest studies exhibit that certain conditions, such as diabetes, prediabetes, and immunosuppression, predispose individuals to HPV infection [25,28]. The persistence of the HPV, especially high-risk types, may be also seen in people with defective immune responses, such as those who are HIV-positive [29,30]. Additionally, HPV is a contributing factor in the pathogenesis of vaginal intraepithelial neoplasia in diethylstilbestrol (DES)-exposed patients [31].

## 5. Pathogenesis and Symptoms of Infection

Undifferentiated basal epithelial cells are infected by the HPV. Nevertheless, access to the lowest cell layer is restricted by the remaining epithelial layers made by fully differentiated cells [32]. The infection occurs through microinjuries that expose deeper layers of the epithelium, allowing the binding of the virion L1 protein to heparin sulfate proteoglycans. In the next step, the capsid changes conformation by cyclophilin B and loses affinity for the primary receptor [33,34]. The N-terminus of the L2 protein is exposed, prone to furin-related proteases. Endosomal escape and infection both require protein cleavage, so lack of interaction with furin prevents escape from the endocytic compartment [35,36,37].

**Figure 1:**
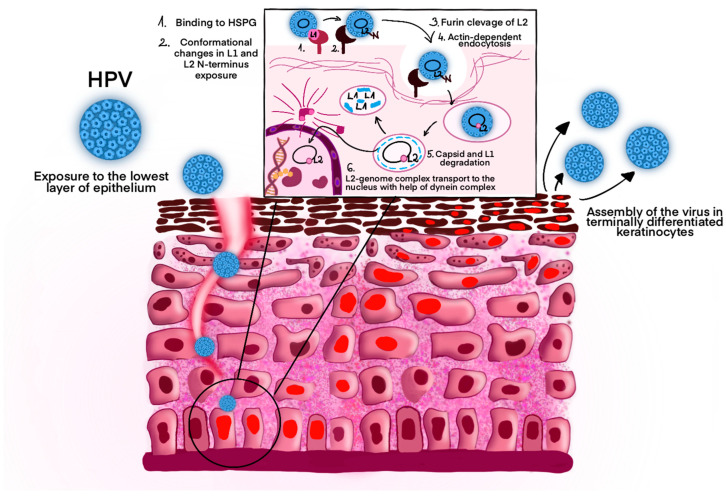
Pathogenesis of HPV infection. Abbreviations: HSPG–heparin sulfate proteoglycans. Original work based on [46]. The graphic shows the process of Human Papillomavirus (HPV) infection in epithelial cells. The virus enters the deepest layer of the epithelium as a result of tissue damage, which allows it to interact with the cells of the basal layer of the epidermis. In the next steps (shown in the diagram), HPV binds to heparan sulfate proteoglycan (HSPG) on the cell surface (1), which induces conformational changes of the capsid and exposure of the N-terminus of the L2 protein (2). This is followed by cleavage of L2 by furin (3) and actin-dependent endocytosis of the virus (4). After internalization, the capsid including L1 protein are degraded (5), and the L2-genome complex is transported to the cell nucleus with help of the dynein complex (6). In the final phase of the replication cycle, the virus is assembled in terminally differentiated keratinocytes and released into the environment.

After many hours of asynchronous internalization of the capsid with the plasma membrane, endocytosis occurs, which is dependent on actin instead of clathrins, lipid rafts, dynamin, or caveolin [31,32]. Virions are transported in endosomes with an acidic environment, where they undergo subsequent structural changes. The L1 protein is directed to lysosomes for degradation, whereas the L2 protein enables the escape of the genome/L2 complex to late endosomal compartments by binding to nexin 17, a sorting protein [32,38]. Moreover, the C-terminus of the L2 protein enables the association with microtubule dynein, which allows the transport of the complex to the nucleus (Figure 1) [39]. Initially, transcription of the E1 and E2 proteins occurs. During the S phase of virus genetic material replication, the E2 protein stabilizes its interaction with DNA, while the E1 acts as an ATP-dependent DNA helicase [40,41,42]. The HPV virus does not have the remaining enzymes necessary for replication, so it uses the host’s enzymes for this purpose. The E6 and E7 proteins can alter the cellular environment in terminally differentiated or growth-arrested cells, which leads to viral genome amplification [32]. As a result of degradation of the pRb protein, the E2F protein is continuously active and binds to DNA transcription factors, leading to the expression of cyclins and other proteins of the cycle. Finally, the virus is assembled in terminally differentiated keratinocytes, where access to oxygen is possible. Disulfide bridges are formed between the L1 proteins, ensuring condensation and stabilization of the capsid [43]. The exit from the cell is mediated by the E4 protein, which binds to cytokeratin filaments [44,45].

Symptoms of primary infection are not noticeable early, as most HPV infections are transient. They resolve after 12–18 months in the case of high-risk HPV infections or after 4–9 months in the case of low-risk HPV infections [47]. The cervicovaginal microbiome (CVM) plays a significant role in the elimination of the virus and the risk of cervical intraepithelial neoplasia 2 or higher (CIN 2/CIN 2+) [48]. A dominance in *Lactobacillus* spp. in CVM is associated with protective role against HPV and decreased risk of HR-HPV-induced CIN lesions [49,50]. In addition, a decreased population of some bacteria leads to an increased number of bacteria associated with the persistence of HR-HPV infections and precancerous lesions, such as *Gardnerella* spp. (*Gardnerella vaginalis*), *Mobiluncus* spp., *Prevotella* spp., *Sneathia amnii*, *Porphyromonas* spp., *Peptostreptococcus* spp., and *Atopobium vaginae* [51]. The findings highlight a significant interplay between vaginal microbiota and HPV, suggesting that microbial dysbiosis could serve as a diagnostic marker or therapeutic target. Monitoring the presence of *Gardnerella* spp. in CMV may allow predicting viral persistence and future cancer risk due to its immunosuppressive properties [3,48]. Specific *Gardnerella* spp. produce sialidase (neuraminidase), encoded by nanH2 and nanH3 genes, which degrade protective vaginal mucosa, detach epithelial cells, and promote biofilm formation [52]. In Novak et al. study women who continue to have cervical HPV16 infection after a year or bacterial vaginosis have higher baseline levels of the nanH3 gene [52]. Furthermore, the amount of *Lactobacillus* spp. may become the prognostic tool of HPV elimination. However, larger prospective studies are needed to validate findings, and applications of innovative microbiome modulation strategies [48,49,51].

The most important symptoms that may indicate an HPV infection include skin warts, *condyloma acuminata*, and recurrent respiratory papillomatosis. Cutaneous warts most often caused by beta and gamma HPV (HPV4, HPV65) are often found in children and young adults, and the peak detection rate reaches 10–14 years of age [3,53]. They are more often observed in males or immunocompromised individuals. Infection most often occurs through skin-to-skin contact. Most of the lesions regress spontaneously within 2 years. In immunocompromised individuals, the risk of developing squamous cell carcinoma is higher [53,54]. HPV6 and 11 are responsible for 90% of condyloma acuminata cases in the anogenital region, on the tongue or lips [55]. The time between the infection and the development of lesions is 11–12 months in men and 5–6 months in women [56]. The possible reason for the duration differences between the development of lesions after HPV infection are immune response, and also anatomical and biological differences, although more focus should be paid to specify the significant ones. Monsonego et al. showed that sex steroid hormones, especially progesterone, may act indirectly on HPV-infected epithelial cells and be implicated as co-factors in HPV-related cervical neoplasia [57]. Ogawa et al. showed that the estrogen/G protein-coupled receptor 30 (GPR30) signaling is involved in proliferation of adenocarcinoma and normal endocervical columnar cells. Additionally, estrogen induced genomic instability, by increasing the number of DNA double strand breaks, leading to carcinogenesis of cervical adenocarcinoma [58]. The lesions appear as flat or small nodules. On moist mucosal surfaces, they may manifest as white or pink, soft growths, while keratinized lesions are characteristic of epithelia with a thick stratum corneum [59]. As a result of HPV16 or HPV18 infection, they may be classified as Bowenoid papulosis, showing condylomatous features with intraepithelial neoplasia. If there is a risk of transformation into cancer, it is possible to remove them during surgery [60].

Recurrent respiratory papillomatosis (RRP) is a benign condition that can develop after an HPV6 or HPV11 infection. It affects the larynx, trachea, pharynx, nasopharynx, nose, oral cavity, and lung parenchyma. It manifests itself with hoarseness, but there is a risk of blocking the airways by the enlargement of the primary cauliflower-like lesion [53,61]. The progressive dysphonia is also an initial symptom in adults [62]. There is a distinction between JoRRP (Juvenile-onset RRP), which is most often acquired perinatally, and AoRRP (Adult-onset RRP), which is associated with oral sex. The first one is very common in Sub-Saharan Africa, while the second one in Europe and South America [53].

## 6. HPV-Related Cancers: Pathogenesis and Epidemiology

**Figure 2:**
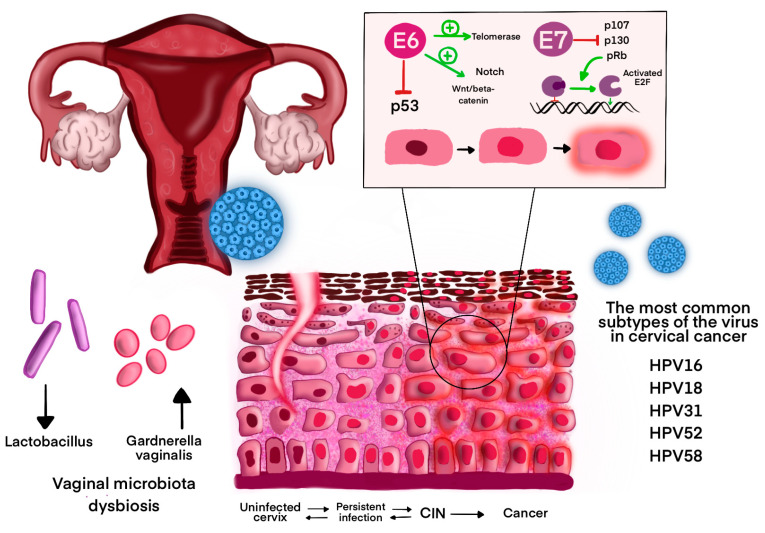
Pathogenesis of HPV-related cervical cancer. Abbreviations: CIN–cervical intraepithelial neoplasia. The graphic shows the mechanism leading from human papillomavirus (HPV) infection to the progression to cervical cancer. After infection of the epithelium, the virus can persist as a chronic infection, leading to pre-cancerous changes (CIN—cervical intraepithelial neoplasia) and then cervical cancer. The oncogenic mechanism of HPV proteins E6 and E7 is shown in the upper right corner. The E6 protein inhibits the action of the tumor suppressor p53 and activates telomerase, supporting uncontrolled cell proliferation. Additionally, it stimulates the Wnt/beta-catenin pathway and the Notch pathway, disrupting cell signaling. On the other hand, E7 deactivates proteins regulating the cell cycle (pRB, p107, p130). Deactivation of pRB protein leads to continuous activation of E2F, dysregulation of the cell cycle promoting the transition from G1 to S phase, and uncontrolled division of epithelial cells. Moreover, the graphic shows the impact of vaginal microbiota dysbiosis on HPV infection. A reduction in the number of protective *Lactobacillus* spp. and an increase in the population of *Gardnerella vaginalis* may promote chronic HPV infection and the progression to cancer. The graphic also shows the most common oncogenic HPV types associated with cervical cancer: HPV16, HPV18, HPV31, HPV52, and HPV58.

The most significant role in the pathogenesis of neoplastic changes resulting from HPV infections is played by the proteins E5, E6, and E7. The E5 protein, present in all types of HR-HPV, except for Betapapillomaviruses, promotes cancer progression by binding to platelet growth factor receptors (PDGFR) and epidermal growth factor receptors (EGFR) [63]. The E6 protein plays a key role in inhibiting the regulation of the cell cycle, as it inactivates the p53 protein. By stimulating the Wnt/β-catenin and Notch pathways, it disrupts normal cell signaling. It also promotes cell immortality by activating telomerase. The E7 protein inhibits the action of pRb, p107, p130. As a result, the E2F factor is activated, which promotes the transition from G1 to S phase (Figure 2) [63,64]. Moreover, in oncogenic HPV types, it binds to the non-receptor protein tyrosine phosphatase PTPN14, leading to its degradation, which results in inhibition of keratinocyte differentiation and their immortality [65]. Both proteins may activate the PI3K/AKT/mTOR and JAK/STAT signaling pathways, which play a role in the pathogenesis of cancer. In particular, JAK/STAT plays an important role in the development of cervical cancer [9,66,67]. These proteins also participate in the inhibition of the immune response. The E5 protein suppresses interferon (IFN) signaling pathway and retains MHC-I molecules in the ER and Golgi Apparatus. The E6 protein has the ability to reduce antigen presentation through a transporter associated with antigen processing complex (TAP) interference. In turn, E7 increases the population of Treg lymphocytes, thereby reducing cytotoxic T, and inhibits pyroptosis. Moreover, both proteins cause up-regulation of immune checkpoint molecules such as PD-L1 [68].

The HPV is associated with 4.5% of all cancers, 8.6% of cancers in women, and 0.8% in men [69]. According to GLOBOCAN data, in 2022, cervical cancer (CC) occurred at a rate of 19.3 per 100,000 women and was the fourth most common cancer in both incidence and mortality in women, with an estimated 660,000 new cases and 350,000 deaths worldwide [6]. The risk of acquiring cervical cancer is 75.4 times higher for women with chronic HR-HPV infections than for women who are HPV-negative, according to a 16-year follow-up study [70]. HPV16 is the most common subtype of the virus in CC globally. The next positions are occupied by HPV18, HPV52, HPV31, and HPV58 [71]. Chronic HR-HPV infection causes cervical illness, which in turn causes cervical cancer. Cervical lesions originate in the transformation zone (TZ), which is situated at the junction between the ectocervix and the endocervix. Low-grade squamous intraepithelial lesions (LSIL) are represented by CIN 1 and high-grade squamous intraepithelial lesions (HSIL) are represented by CIN 2 and CIN 3. The replication activity decreases as the grade of lesion increases [68].

Cervical cancer is not the only cancer associated with HPV infection. Vulvar cancer and vaginal cancer are associated with HPV in 70% and 75%, respectively [72]. Moreover, HPV viruses cause about 26–30% of head and neck cancers, where the incidence of HPV-related oropharyngeal squamous cell carcinoma (OSCCC) is about 30% (data from 2020) and is constantly increasing, especially in developed countries [73]. In the last two decades in some European countries, the incidence has increased significantly [30,74]. Women have a higher incidence of anal cancer and precancerous anal lesions than men, and 90% of these lesions are related to HPV16, 18, and 33 [72,75]. The incidence is particularly high in men who have sex with men (MSM), especially those with HIV [76,77]. Penile cancer is related to HPV in 60% of cases [72]. Moreover, this virus can be detected in 79.8% of cases of penile intraepithelial neoplasia (PeIN) and 90% of genital warts cases. The most frequently documented subtype was HPV16 [53]. It has been demonstrated that HR-HPV found in benign prostatic tissues immortalizes prostate cells, which could be connected to the gradual transformation of the prostate gland from a benign neoplasm to prostate cancer [78,79].

## 7. World Health Organization Guidelines—HPV Vaccine

Currently there are six licensed HPV vaccines (Table 1). In 2020, the World Health Organization (WHO) updated its position on human papillomavirus (HPV) vaccines, introducing new recommendations that included the option of a single-dose schedule [80]. This approach, endorsed by the WHO’s independent advisory group SAGE in April 2020 [81], offers efficacy and protection comparable to the traditional two- or three-dose regimen. The update comes amidst alarming global declines in HPV vaccination rates. Between 2019 and 2021, the proportion of girls receiving the first dose of the vaccine dropped from 25% to 15%, leaving an additional 3.5 million girls unvaccinated in 2021 compared to 2019 [82].

The revised guidelines aim to enhance vaccine accessibility by simplifying vaccination logistics, reducing the costs and challenges associated with follow-up appointments, and enabling countries to vaccinate a larger number of girls. The WHO emphasizes the urgent need to strengthen HPV vaccination programs, implement these updated schedules swiftly, and reverse recent declines in coverage [82].

In 2022, further evidence confirmed that a single HPV vaccine dose could provide strong protection against persistent HPV infection [84]. This prompted the WHO to recommend a flexible approach, allowing countries to adopt either a one- or two-dose schedule for key target groups:

- Girls aged 9–14 years: one or two doses.
- Young women aged 15–20 years: one or two doses.
- Women over 21 years: two doses spaced 6 months apart.

Special guidelines prioritize individuals who are immunocompromised or living with HIV. These individuals should receive at least two doses and, when feasible, three doses for optimal protection [85].

The WHO continues to advocate for vaccinating girls aged 9–14 years during early adolescence, ideally before sexual activity begins. Focusing on girls not only directly prevents cervical cancer but also offers herd immunity to boys when coverage is high. This strategy is more cost-effective than vaccinating both sexes, although some regions choose to include boys in their programs [82].

In low- and middle-income countries (LMICs), school-based vaccination programs have proven particularly effective, achieving higher coverage rates than healthcare facility-based efforts. Countries that have achieved widespread HPV vaccination among adolescent girls have documented significant decreases in HPV prevalence, cervical precancers, and invasive cervical cancers. These successes underscore the critical role of robust vaccination programs in combating HPV-related diseases globally [82].

## 8. World Health Organization—HPV Vaccine News 2024

In October 2024, the World Health Organization (WHO) announced the prequalification of Cecolin as the fourth human papillomavirus (HPV) vaccine approved for use in a single-dose schedule [86]. This decision was supported by new data demonstrating that Cecolin meets the criteria established in the WHO’s 2020 guidelines for alternative, off-label single-dose HPV vaccination. This milestone is expected to enhance the global supply of HPV vaccines, making it possible to vaccinate more girls and reduce the burden of cervical cancer.

The Cecolin HPV vaccine, a domestic bivalent vaccine targeting HPV types 16 and 18, was introduced in China in 2020 at a lower price than imported alternatives [87]. Clinical trials have demonstrated its safety and immunogenicity, with non-inferiority to Gardasil in a two-dose schedule [88]. A phase 2 trial of a nonavalent version, Cecolin 9, showed high seroconversion rates and good tolerability in women aged 18–45 [89]. A network meta-analysis comparing HPV vaccines found that Cecolin was most effective against HPV 18 persistent infection, with 98% efficacy [90]. However, public perception varies, with women in economically developed areas preferring imported vaccines due to concerns about effectiveness and quality [87]. Increasing awareness and education about the domestic vaccine, particularly in underdeveloped areas, could help prevent cervical cancer and improve vaccine uptake [87].

In addition, on 2 August 2024, the WHO prequalified a fifth HPV vaccine, Walrinvax, for global use. This vaccine is approved with a two-dose schedule, further strengthening the supply of HPV vaccines and expanding access for girls in need of protection. A study on this bivalent HPV16/18 vaccine demonstrated non-inferior immune responses in adolescent girls compared to young women, with persistent antibody levels up to 36 months post-vaccination [91]. While Walrinvax is not currently recommended for single-dose use, additional research may determine its suitability for this approach in the future.

These developments represent significant progress in efforts to ensure sustainable HPV vaccine availability worldwide, ultimately supporting expanded vaccination programs to prevent cervical cancer on a larger scale.

## 9. The HPV Vaccination Strategy: The Example of Poland

The universal HPV vaccination program in Poland implements the assumptions and goals of the National Oncology Strategy for 2020–2030. It supplements the free Vaccination Program for children and adolescents with a new scope of protection against diseases caused by HPV. The vaccines available under the program are 2-valent Cervarix and 9-valent Gardasil 9. As of 1 September 2024, free-of-charge vaccinations under the universal HPV vaccination program are available for children from the age of 9 to the age of 14. Vaccinations are administered in two doses. The interval between these doses in the program is from 6 to 12 months [92]. Vaccination guidelines are aligned with the WHO recommendations (Table 2).

This marks a significant shift from the previous system where HPV vaccines were recommended but not publicly funded [94]. The first program launched in 2023. Prior to this program, HPV vaccination rates were low, with only 16% of adolescents reporting being vaccinated [95]. A study conducted shortly after the program’s launch found that 51.3% of adults were aware of the free vaccination program, with television being the primary source of information [96]. The same study revealed that 63.3% of respondents were willing to vaccinate their children against HPV. Factors associated with higher awareness and willingness to vaccinate included female gender, higher education, and living in large cities [96]. These findings highlight the importance of education and accessibility in improving HPV vaccination rates in Poland.

## 10. Self-Sampling for HPV Testing

Cervical cancer can also be prevented through screening that detects precancerous changes in the cervix, allowing for their treatment before they develop into cancer. In the USA, women’s health professionals usually run a combination of Pap and HPV tests. But, the new self-collection methods that have been approved can help women collect their samples by themselves in a range of healthcare settings, which may include primary care, mobile health clinics, etc. This development offers a great opportunity to improve access and provide women with a more acceptable option to keep up with regular screenings [97].

With HPV self-sampling, there is no need for a cervical exam. It collects vaginal material via swabs, tampons, or brushes, which are analyzed for high-risk HPV strains [98]. Evidence from randomized trials has shown that HPV testing is a more effective screening method for cervical cancer than cytology [99]. Recent studies indicate that self-collected vaginal samples for high-risk HPV testing are as sensitive as cytologic examination of clinician-collected cervical samples. The former process is, however, less specific [98]. Plus, studies from randomized trials conducted internationally show that sending out self-sampling kits increases participation in screening among under-screened women as compared to clinician-collected samples [100]. Even so, some women prefer clinician-collected samples because they are concerned about their ability to collect a reliable sample on their own [101]. Right now, we need more research to understand how this affects screening frequency and if it causes any social harm or adverse outcomes.

## 11. Screening Tests in the Diagnosis of HPV Infections

Recent studies on HPV screening in Poland reveal important insights into cervical cancer prevention. Type-specific HPV DNA screening should focus on types 16, 18, and 45 [102,103]. A large-scale study using liquid-based cytology, HPV testing, and p16/Ki67 dual-staining demonstrated reporting rates consistent with international standards, indicating the potential effectiveness of private-based screening models [104]. Self-reported participation in cervical cancer screening has improved significantly, with 86.7% of Polish women aged ≥15 years declaring they had undergone a Pap test by 2019. However, disparities in screening rates persist across age groups, education levels, urbanization, incomes, and regions, highlighting the need for targeted interventions to address low participation in specific demographics [105].

The free cytology program in Poland was launched 1 November 2023. It covers women aged 25 to 64. The main goal is to reduce the number of women who get sick and die from cervical cancer. A woman can have a cytology if she has not had one in the last three years. She can also have a test after a year if she has risk factors such as HIV infection or HPV or is taking immunosuppressive drugs. Woman can have a test under the program free of charge if they are entitled to healthcare services [106].

## 12. The Role of Colposcopy in Cervical Cancer Screening—New Polish Recommendations

Recent studies have examined the role of HPV testing and colposcopy in cervical cancer screening. HPV testing has shown higher sensitivity than colposcopy in detecting cervical intraepithelial neoplasia (CIN), while colposcopy demonstrates better specificity. Combining HPV testing and colposcopy appears to be the most effective method for CIN detection [107].

The 2020 recommendations of the Polish Society of Gynecologists and Obstetricians, along with the Polish Society of Colposcopy and Cervical Pathophysiology, present an updated approach to colposcopic terminology in Poland. These guidelines were developed based on the 2011 nomenclature created by the International Federation for Cervical Pathology and Colposcopy (IFCPC) and seek to standardize practices and maximize clarity, comparability, and global harmonization.

Colposcopy is an integral part of cervical cancer screening programs worldwide, having a particular role in the detection of HSIL and cervical cancer. These include the HPV-dependent model of screening for secondary cervical cancer and terminological advances from the LAST (Lower Anogenital Squamous Terminology) project.

Notably, the 2020 colposcopy terminology updates suggest the addition of the terms “adequate” or “inadequate colposcopy,” replacing “satisfactory” or “unsatisfactory colposcopy,” which were used previously [108]. This shift highlights the diagnostic utility of the exam rather than simply the visibility of the structures. Furthermore, additional diagnostic features have been added to aid in recognizing colposcopic lesions. For example, the “sign of the inner border” means a sharp boundary between the narrow and wide whitening area, whereas the “ridge sign” indicates a transparent, elevated folding in the transition zone. These updates enhance the prediction and detection of HSIL.

The guidelines also refine the classification of colposcopic findings. Whether the examination is adequate will depend on if it allows for diagnostic assessment. It is inadequate if it is hindered by factors such as inflammation, bleeding, or scarring. The squamocolumnar junction (SCJ) is graded as fully visible, partially visible, or not visible. The scientific name of these cervix regions is the transformation zone, and it is categorized into three types: Type 1 (entire visible), Type 2 (partially visible but achievable), and Type 3 (not fully visible) [108]. Colposcopic findings that can be normal are mature squamous epithelium, ectopia, and Nabothian cysts, as well as openings of the glands. Abnormal findings are categorized as either minor changes consisting of thin acetowhitening and fine mosaic patterns or major changes consisting of dense acetowhitening, coarse mosaics, and punctation. Lugol’s iodine staining, previously fitted into the minor finding category, has now been categorized as a nonspecific change because of its predictive value for HSIL.

These recommendations go further to incorporate clinical and legal implications, highlighting the importance of the standardization of terms in order to achieve accuracy, ensuring consistency from cytology to histology and colposcopy. It has been brought out that accurate documentation will reduce medical–legal risks and improve communication among clinicians [108].

## 13. Treatment of HPV-Related Cervical Cancer

Depending on the cervical cancer stage at the time of diagnosis, the primary treatment options with curative intent are surgery, chemoradiation, and a combination [109]. The presence of a cancer subtype (Squamous cell carcinoma (SCC), adenocarcinoma (AC), adenosquamous carcinoma (ASC)) or HPV infection does not exclude any treatment option [109]. Three groups were distinguished to describe clinical management and will be used to describe the latest updates in treatment, relating to Féderation Internationale de Gynécologie et d’Obstétrique (FIGO) staging:

1. Early-Stage Cervical Cancer (FIGO IA-IIA)
2. Locally Advanced Cervical Cancer (LACC) (FIGO IIB–IVA, extending to the pelvic side wall or adjacent organs)
3. Recurrent or Metastatic Cervical Cancer (FIGO IVB, any T any N, M+) [109,110,111].

The summary of treatment information is presented in Table 3.

### 13.1. Early-Stage Cervical Cancer (FIGO IA–IIA)

The treatment of choice in this stage is surgery, most commonly radical hysterectomy combined with systematic pelvic lymphadenectomy with or without sentinel lymph node (SLN) [112]. SLN dissection should be considered in FIGO stage I patients with tumors of ≤4 cm only in highly specialized centers. It is performed by injecting a tracer directly into the cervix and using blue dye, technetium radiocolloid, or fluorescent indocyanine green. Moreover, SLN should be detected on both sides. [111]. Microinvasive cervical cancer (stage IA1) without lymphovascular space invasion (LVSI) can be treated with preservation of fertility by conization or simple trachelectomy [111]. In the 2020 update in the clinical guidelines, ESMO stated the fact that radical hysterectomy performed by laparoscopy or robot-assisted surgery cannot be regarded as the preferred treatment in comparison with open surgery [111,113,114]. In the ESGO2023 guidelines, radical hysterectomy in the form of minimally invasive surgery may be considered only in low-risk tumors (<2 cm and free margins after conization) and in high-volume centers only with the patient’s consent [115]. The possibility of ovarian preservation should be considered, especially in young women with HPV-related cervical cancer, but it is not recommended in cases of HPV-independent adenocarcinomas [112]. If the ovaries are preserved, opportunistic bilateral salpingectomy or exclusive radio-chemotherapy should be added to the treatment [115]. When we consider de-escalation from radical to simple hysterectomy in cervical cancer tumors ≤2 cm, with the possible benefits and risks, we should point out that recent studies confirmed that simple hysterectomy is comparable to radical in terms of the 3-year incidence of pelvic recurrence [116,117,118]. In contrast to the previously mentioned recommendations, the 20-year randomized study showed that there was no advantage to surgery compared to radiation in treatment for early-stage cervical carcinoma in terms of survival, and the treatment should be chosen by considering clinical factors such as menopausal status and comorbidities. Nevertheless, for histological-type adenocarcinoma, surgery was confirmed as a better choice [132]. Expert opinions suggest that for postmenopausal patients with comorbidities, radical radiotherapy could be a safer option in cases of FIGO I-II, but there is no strong evidence [110].

### 13.2. Locally Advanced Cervical Cancer–LACC (FIGO IIB–IVA)

For many years, based on randomized phase III trials, the gold standard for treating LACC was invariably platinum-derived (cisplatin alone [preferred] or cisplatin/fluorouracil) chemotherapy and concomitant external radiation therapy followed by brachytherapy [112,119,120,121,122,123].

In the case of a cisplatin-intolerant patient, NCCN guidelines recommend to use carboplatin; alternatively, capecitabine/mitomycin, gemcitabine, and paclitaxel can be considered when cisplatin and carboplatin are unavailable [109]. The NCCN emphasizes that the cost and toxicity profiles of radiosensitizing agents should be considered when selecting an appropriate regimen for treatment, especially when these regimens are being used for extended-field RT where toxicities may be more severe [109]. The INTERLACE trial (2012–2022)was a multicenter, randomized phase 3 trial conducted at 32 medical centers, including patients with locally advanced cervical cancer (FIGO stage IB1 disease with nodal involvement, or stage IB2, IIA, IIB, IIIB, or IVA), to compare standard cisplatin-based chemoradiotherapy (once-a-week intravenous cisplatin 40 mg/m^2^ for 5 weeks with 45.0–50.4 Gy external beam radiotherapy delivered in 20–28 fractions plus brachytherapy to achieve a minimum total 2 Gy equivalent dose of 78–86 Gy) alone to induction chemotherapy (once-a-week intravenous carboplatin area under the receiver operator curve 2 and paclitaxel 80 mg/m^2^ for 6 weeks) followed by standard cisplatin-based chemoradiotherapy [124]. The findings of this study revealed that short-course induction chemotherapy followed by chemoradiotherapy significantly improved the survival of patients with LACC, regardless of the response to induction chemotherapy [124]. In contrast to the benefits of induction before chemoradiotherapy, the OUTBACK study showed that four cycles of adjuvant paclitaxel 155 mg/m^2^ plus carboplatin AUC 5 dosed 3 times a week did not improve 5-year OS compared to chemoradiotherapy alone (72% vs. 71%, HR = 0.90; 95% CI = 0.70–1.17; *p* = 0.81) [125]. Another phase 3 study (3 ENGOT-cx11/GOG-3047/KEYNOTE-A18 clinical trial) for LACC (FIGO IB2–IIB node-positive and stage III–IVA any nodal status) treatment assessed the efficacy and safety of adding to the standard chemoradiotherapy an anti-programmed death-1 (anti-PD-1) immune checkpoint inhibitor (ICI)—pembrolizumab. This study compared two groups of patients treated with five cycles of pembrolizumab (200 mg) or placebo every 3 weeks, plus chemoradiotherapy, followed by 15 cycles of pembrolizumab (400 mg) or placebo every 6 weeks [126]. The results revealed that the addition of pembrolizumab to the conventional chemoradiotherapy significantly improved progression-free survival in patients with newly diagnosed, high-risk, locally advanced cervical cancer [126]. The third interesting recent study for LACC treatment—the CALLA trial — assessed the efficacy of adding durvalumab, a PD-L1 antibody, with and following chemoradiotherapy [127]. The findings suggest that durvalumab did not significantly improve progression-free survival in a biomarker unselected, all-comers population, but the authors suggest its further exploration in patients with high-tumoral PD-L1 expression [127]. In summary, adding induction chemotherapy (INTERLACE for FIGO stages IB3–IVA, except IIIA and those with nodal disease above the aortic bifurcation), and, separately, concurrent-maintenance pembrolizumab (KEYNOTE-A18 trial in FIGO stages IIIA–IVA), to the standard chemoradiotherapy created two new, more effective treatment protocols for LACC, from witch INTERLACE has more potential for worldwide application in LACC than KEYNOTE-A18, because induction chemotherapy has already demonstrated a 39% relative risk reduction of all-cause mortality compared to the standard chemoradiotherapy alone [128].

### 13.3. Recurrent or Metastatic Cervical Cancer (FIGO IVB)

In cases of recurrent or metastatic cervical cancer, the treatment of choice is palliative chemo- or chemoradiotherapy [109]. According to the NCCN, the preferred first-line chemotherapy is the combination of platinum-based chemotherapy (cisplatin or carboplatin/paclitaxel/bevacizumab), because very often those patients who develop metastasis have already received concurrent cisplatin/RT and may not respond to single-agent platinum therapy [109].

The NCCN also recommends platinum-containing combination regimens are cisplatin/paclitaxel (category 1), carboplatin/paclitaxel (category 1), topotecan/paclitaxel/bevacizumab (category 1), topotecan/paclitaxel, and cisplatin/topotecan as appropriate alternative options for certain patients [109].

New treatment options are also included in the newest recommendations of many scientific societies. The KEYNOTE-826 study revealed the efficacy of pembrolizumab combined with chemotherapy (with or without bevacizumab), with clinically meaningful improvements in OS, and, therefore, it is now a category 1 preferred treatment for patients with PD-L1 (CPS ≥1) tumor expression. [109,112,115,128,129].

Another open-label, randomized trial proposes a new first-line therapy option. BEATcc (ENGOT-cx10/GEICO 68-C/JGOG1084/GOG-3030) compared the anti-PD-L1, atezolizumab, paclitaxel–platinum plus bevacizumab regimen and proved that the addition of atezolizumab to a standard bevacizumab plus platinum regimen significantly improves progression-free and overall survival [130].

In cases of progression after first-line chemotherapy, unfortunately, progression-free survival was approximately 3 to 6 months [131].

Second-line therapy for recurrent or metastatic cervical cancer options depend on the tumors’ characteristics and include:

- chemotherapy (paclitaxel, albumin-bound paclitaxel, docetaxel, fluorouracil, gemcitabine, pemetrexed, topotecan, vinorelbine, and irinotecan),
- bevacizumab,
- pembrolizumab,
- tisotumab vedotin-tftv,
- cemiplimab,
- Nivolumab for PD-L1–positive tumors,
- Selpercatinib for RET gene fusion–positive tumors,
- TRK inhibitors for NTRK gene fusion–positive tumors (larotrectinib, entrectinib),
- Trastuzumab deruxtecan for HER2-positive tumors,
- biomarkers with their associated targeted treatments as second-line/subsequent therapies [109].

## 14. Future Directions

Perspectives for new diagnostic and treatment methods:

### 14.1. Therapeutic Vaccine

In a different strategy than preventive vaccines, which result in neutralizing antibodies, therapeutic vaccines aim to generate cell-mediated immunity [133]. The main targets are oncoproteins E6 and E7, as they play a crucial role in the development of the malignancies associated with HPV and are expressed in precancerous and cancerous lesions. There are various types of vaccines tested in clinical trials with promising results, which are based on

- bacterial vector (*L. monocytogenes*, *L. lactis*, *L. plantarum*, *L. casei*, Salmonella, *Shigella*, and *E. coli*),
- viral vector (adenoviruses, adeno-associated viruses, alphaviruses, and vaccinia virus),
- peptide,
- protein,
- DNA,
- RNA replicon,
- dendritic cell,
- tumor cell [133].

Adoptive T-cell therapy is another strategy that offers more rigorous control over the magnitude of the targeted response than tumor vaccination, and it showed clinical activity for LACC in phase-II-trial patients [134,135].

Moreover, Kim et al. conducted a study on electroporation-enhanced immunization with a rationally designed HPV DNA vaccine (GX-188E) that targeted HPV antigens to dendritic cells, eliciting a significant E6/E7-specific IFN-γ-producing T-cell response in all nine cervical intraepithelial neoplasia 3 (CIN3) patients [136].

Another recent study presented the safety and efficacy of a dual-purpose (targeting active infections as well as established HPV-related malignancies) HPV nanoparticle vaccine (cPANHPVAX) with eight different HPV L2 peptide epitopes on the E7 oncoantigens from HPV16 and 18. This new solution can be beneficial for uninfected and infected patients, acting as both a preventive and curative agent [137].

In conclusion, the combination of HPV therapeutic vaccines with radiotherapy, chemotherapy, immunomodulators, or immune checkpoint inhibitors creates new improvement potential in cervical cancer treatment [135].

### 14.2. Oncoproteins E6 and E7 Antibodies as Serological Markers

Another method that was assessed in a meta-analysis is HPV16 and HPV18 E6 and E7 antibodies, and its findings suggest that those markers have high specificity but low sensitivity to detect cervical cancer or precancerous lesions [138]. A recent meta -analysis stated in its conclusions that this method is suitable to be used for triaging HPV-positive women because of its high specificity; nevertheless, oncoprotein-negative women would not be recommended to undertake routine screening, as they are in need of follow-ups [139].

### 14.3. Circulating Cell-Free DNA (cfDNA) 

A recent study reported on a blood-based test for HPV-specific E7 and L1 genes, measured in cell-free DNA (cfDNA) extracted from plasma by the use of droplet digital PCR [140]. This diagnostic method may serve as a tumor marker to guide treatment and detect early recurrence in cervical cancer, because the statistics showed significant correlation between high viral load (defined as ≥20 E7 or L1 copies per 20 μL reaction volume) and increased risk of recurrence and death at 5 years on univariate analysis but not multivariate analysis [140]. In addition, another study had results that confirmed a minimally invasive method of detecting HPV E7 cfDNA as a potential tumor marker, with great specificity and moderate sensitivity for monitoring cervical cancer [141].

## 15. Conclusions

Since HPV was identified as the necessary cause of cervical cancer, HPV-based technologies have become central to innovative strategies for both primary and secondary prevention. These advancements include the use of HPV testing in screening programs and the introduction of HPV vaccines for preadolescent girls and young women. When implemented broadly and strategically, these protocols have the potential to fulfill Papanicolaou’s vision of eradicating cervical cancer by extending the benefits of prevention to populations in developing regions worldwide. With the continuous advancement of medical technologies, faster and more convenient methods for conducting such tests are becoming available. However, the primary strategy for combating cervical cancer remains testing and vaccination. New clinical trials explore the addition of immunotherapy and new protocols with induction therapy to the treatment of higher-staged cervical cancers with promising results for future applications.

## Figures and Tables

**Table 1:** WHO-licensed HPV vaccines 2024 [83].

Brand Name [83]	Valency	Target–HPV Types
Cervarix	bivalent	16, 18
Cecolin	bivalent	16, 18
Walrinvax	bivalent	16, 18
Gardasil	quadrivalent	16, 18
Cervavax	quadrivalent	16, 18, 6, 11
Gardasil9	nonvalent	16, 18, 31, 33, 45, 52, 58, 6, 11

**Table 2:** Vaccination guidelines in Poland, 2024 [93].

Age at the time of commencement of vaccination	2-valent vaccine	4-valent vaccine 9-valent vaccine
9–14 years old	2-dose regimen second dose 5 to 13 months after the first dose	2-dose regimen second dose 5 to 13 months after the first dose
	if the second dose of vaccine is administered earlier than 5 months after the first dose, administer the third dose	
15 years old and older	3-dose regimen (0, 1, 6 months)	3-dose regimen (0, 2, 6 months)

**Table 3:** Treatment of HPV-related cervical cancer updates.

FIGO Stage	Guidelines	New Options	Additional Information	References
FIGO IA	Trachelectomy			[111]
FIGO IB-IIA	Radical/simple hysterectomy		-laparoscopy or robot-assisted surgery cannot be regarded as the preferred treatment in comparison with open surgery -radiotherapy could be safer than surgery for postmenopausal patients	[111,112,113,114,115,116,117,118]
FIGO IIB-IVA	Platinum-derived chemotherapy and concomitant external radiation therapy followed by brachytherapy	-addition of induction chemotherapy -addition of pembrolizumab to the conventional chemoradiotherapy	-exploration of durvalumab use in patients with high tumoral PD-L1 expression	[109,112,119,120,121,122,123,124,125,126,127,128]
FIGO IVB	Palliative chemo/chemoradiotherapy (combination of platinum-based chemotherapy) Second-line therapy	-pembrolizumab combined with chemotherapy (+/−bevacizumab) -addition of atezolizumab to a standard bevacizumab plus platinum regimen	-second-line therapy choice depends on tumor characteristics	[109,112,115,128,129,130,131]
